# Surface Charge Dependence of the Direct Piezoelectric Response of a Room Temperature Ionic Liquid: Implications for Nanoscale Control over Sensing and Actuation

**DOI:** 10.1021/acsanm.6c00579

**Published:** 2026-04-01

**Authors:** Neelanjana Mukherjee, Sheryl S. Blanchard, G. J. Blanchard

**Affiliations:** Department of Chemistry, 3078 Michigan State University, 578 S. Shaw Lane, East Lansing, Michigan 48824 United States

**Keywords:** ionic liquid, piezoelectric response, surface charge, monolayer growth, monolayer charge

## Abstract

We report on the surface charge-dependence of the direct piezoelectric response of a room temperature ionic liquid (RTIL). Surface charge is controlled chemically by the surface-modification of ITO-coated glass supports with a nanometer thick monolayer of graphene oxide phosphate (P-GO), which carries a net negative charge when phosphate terminated and a net positive charge upon reaction with Zr^4+^. The dependence of the RTIL direct piezoelectric response on the surface charge correlates with the ability to modulate the surface-induced charge density gradient seen in RTILs, establishing an empirical connection between the direct and converse piezoelectric responses in RTILs.

## Graphical abstract

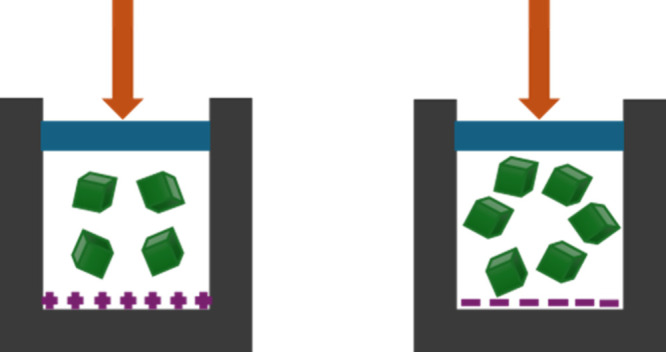

Room temperature ionic liquids (RTILs) comprise a class of compounds that coexist as associated and dissociated cations and anions, which leads to useful properties that differ from molecular liquids, such as a characteristically wide electrochemical window, extremely low volatility, and the absence of flammability. For these reasons, RTILs have proven to be useful in a variety of technological applications,^1,2^ including supercapacitors, lithium batteries, fuel cells, catalysis, chemical separations, ionogels, and lubrication.^3–6^ Because RTILs exist in the liquid phase at ambient temperature and pressure, there has been a historical tendency to understand their properties in the same framework as that used for molecular liquids, and this approach has not been able to account for RTIL behavior on a variety of length- and time scales. For example, we recently reported the direct piezoelectric effect in RTILs,^7–9^ which arises from a pressure-induced liquid-to-crystalline solid phase transition. While much work needs to be done to understand this novel phenomenon at the molecular and microscopic levels, it is known that the direct piezoelectric response of RTILs can be influenced by surface templating.^10^ Related to these findings, there have been numerous reports of nascent organization in RTILs which is influenced by the presence and properties of the solid interface the RTIL is in contact with, and the organization has been reported to persist over length scales ranging from nm to tens of μm, depending the means used to probe the organization.^11–15^ The presence of a surface-induced charge density gradient, *ρ*_*f*_, in RTILs has been identified as the converse piezoelectric effect, but the connection between the direct and converse piezoelectric responses remains to be made. The complicating factor in making this connection is that RTILs exist in the liquid phase under ambient conditions, and the model used to describe the piezoelectric effect was developed for solid materials. The direct piezoelectric effect in RTILs occurs upon the application of pressure to produce a transient crystalline solid, which is the actual piezo-active material, and a consequent charge separation. Understanding the converse piezoelectric effect, where the application of charge causes a physical distortion, is somewhat more complicated because for liquids, only the compression modulus is comparable to that of solids, and both Young’s modulus and the shear modulus are vastly different for liquids and solids. The converse piezoelectric response in RTILs is seen as the ability to induce a free charge density gradient, *ρ*_*f*_, by exposure of the RTIL to a charged interface, and the spatial extent of *ρ*_*f*_ is on the order of 50 μm. It is known that *ρ*_*f*_ can be controlled by the sign of the support surface charge,^16^ and it is therefore of interest to determine whether or not the direct piezoelectric response correlates with the converse piezoelectric response. It is the purpose of this communication to understand this relationship more directly.

The organization seen in RTILs over length scales ranging from nanometers to tens of μm^11–15^ has resisted a comprehensive understanding, to some extent because a variety of different means have been used to examine said organization, but in essentially all cases, the range of the organization is vastly in excess of that predicted by models for molecular liquids, such as the Gouy–Chapman or Gouy–Chapman-Stern models. A very recent report by Lasar et al. has provided significant insight into interfacially induced organization in RTILs, which likely provides the basis for the connection between the direct and converse piezoelectric responses in RTILs.^13^ In that work, the authors showed that ordered RTIL domains formed at interfaces where the domains were on the order of 400 to 700 nm, and this dimension corresponds closely to the characteristic dimension of the pressure-induced transient crystal structures estimated previously.^9^ The implication of this correspondence is that the interfacial domains may be “seeds” for the formation of piezo-active crystals. We demonstrated recently a relationship between interfacial structural modification with a molecular monolayer (∼2.3 nm thick) and the magnitude of the RTIL piezoelectric response.^10^

Prior work demonstrating the existence of *ρ*_*f*_ in RTILs revealed that the sign of *ρ*_*f*_ could be controlled by the sign of interfacial charge.^14,16^ It is thus of interest to determine whether interfacial charge can have an influence on the direct piezoelectric response of RTILs. We have used a pressure vessel where the head of the cylinder in contact with the RTIL is Indium Tin Oxide (ITO)-coated glass,^10^ and we have modified this surface by depositing a layer of phosphated graphene oxide (P-GO)^17^ to impart a negative net charge. We change the surface charge of this interface by reacting it with Zr^4+^ using well-established chemistry.^17–20^ We find that the direct piezoelectric response of the RTIL *N*-butylpyridinium bis­(trifluoromethyl-sulfonyl)­imide (C_4_Py TFSI) depends on the sign of the surface charge. The piezoelectric response of C_4_PY TFSI is increased slightly when in contact with the negatively charged P-GO surface and, decreased slightly when in contact with the positively charged P-GO surface. This is the first report of surface charge-dependent direct piezoelectric response for a RTIL and provides correspondence to earlier measurements of RTIL converse piezoelectric response.

The room-temperature ionic liquid *N*-butylpyridinium bis­(trifluoromethyl sulfonyl)­imide (C_4_Py TFSI) (Figure 1) was purchased from Sigma-Aldrich and purified prior to use according to a procedure reported previously.^14,21^ This RTIL was used because of its favorable piezoelectric properties and ready availability. Zirconyl chloride octahydrate (ZrOCl_2_·8H_2_O, 98%), anhydrous acetonitrile (CH_3_CN anhydrous, 99.8%), phosphorus oxychloride (POCl_3_, 99%), collidine (99.8%), methanol (99.8%), isopropanol (>99.5%) and ethanol (>99.5%) were purchased from Sigma-Aldrich. Phosphated graphene oxide (P-GO) was synthesized according to the procedure reported previously.^17^ All reagents were used as received, without further purification. Ultrapure Milli-Q water (18 MΩ) was supplied by a Thermo Scientific Genpure system and used in all experiments. The ITO coated glass discs were purchased from Nanocs Inc. (IT10–111–25, 10 Ω sq^–1^). The glass discs were cleaned by immersion in Milli-Q water and detergent (Fisher Sparklin 1) and sonicated for 15 min, then rinsed with Milli-Q water to remove detergent, immersed in Milli-Q water and sonicated for 15 min followed by isopropanol for 15 min. After rinsing with ethanol, the ITO-coated discs were stored in Milli-Q water prior to use.

**Figure 1:**
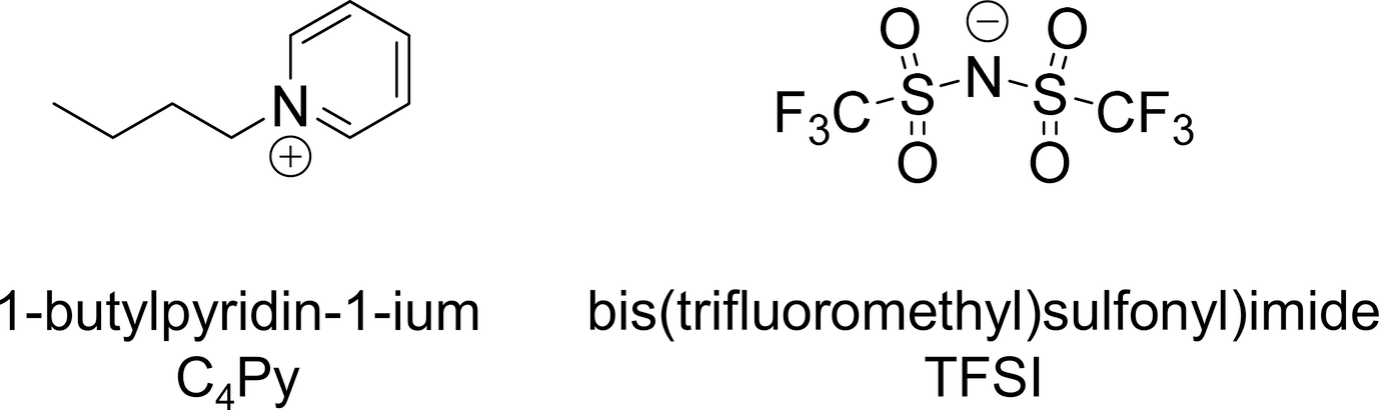
Chemical structure of the ionic liquid used.

The cleaned ITO discs were dried using a stream of N_2_(*g*) and were phosphated directly using POCl_3_ and collidine in anhydrous acetonitrile in a fume hood.^17^ After 10 min, the substrates were rinsed with Milli-Q water and dried under a stream of N_2_(*g*). The discs were zirconated by immersion in a 5 mM aqueous ethanol solution of ZrOCl_2_ (60% ethanol, 40% water v/v) for 5 min. For adlayer formation, the zirconated discs were immersed in the P-GO solution (∼0.2 M in CH_3_CN) for 10 min to produce a negatively charged surface. For positively charged surfaces, the same deposition procedure was used to produce a P-GO monolayer, followed by immersion in the Zr solution for 5 min to produce a Zr^4+^-terminated monolayer (Figure 2). Reacted discs were dried under a stream of N_2_(*g*) and then placed in an oven (110 °C) overnight before use. The characterization of these monolayers by optical ellipsometry, absorbance, XPS and SEM has been reported in detail previously.^17^

**Figure 2:**
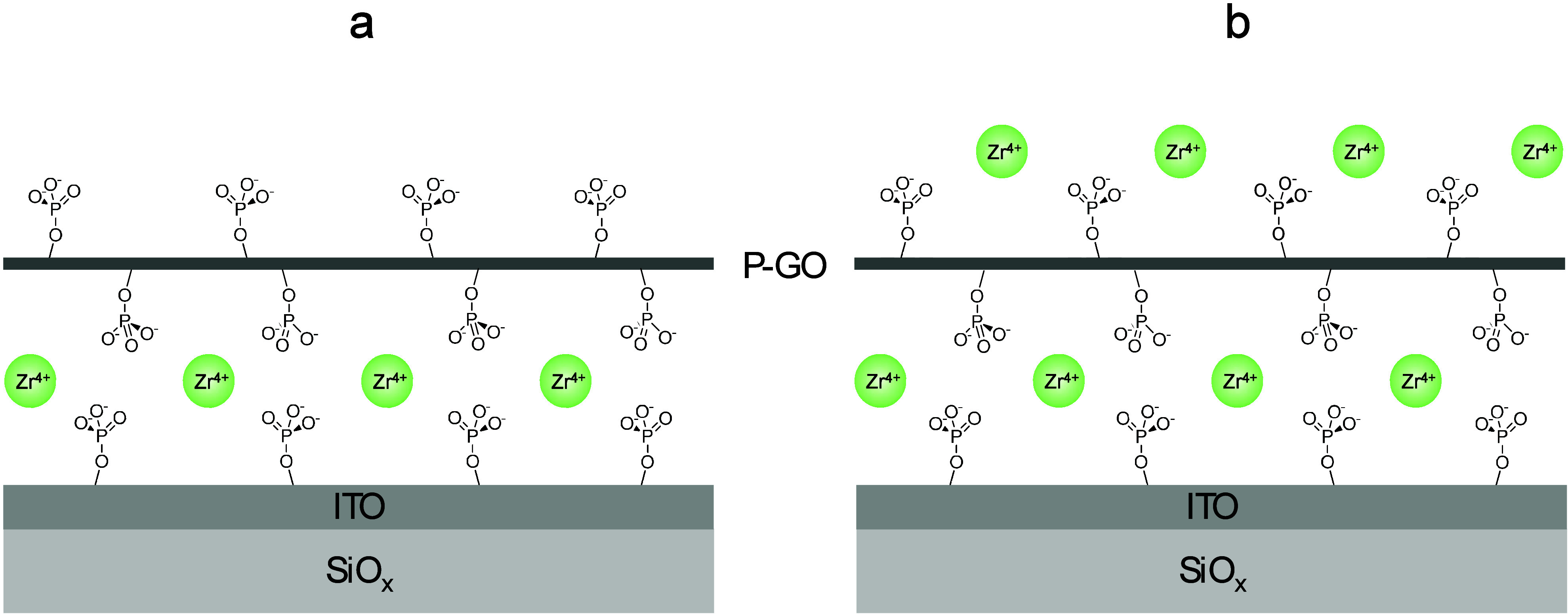
Schematic of monolayers formed on the ITO surface. (a) The modified surface is negatively charged (P-GO). (b) The modified surface is positively charged (Zr).

Measurement of the magnitude of the direct piezoelectric effect was performed using an instrument designed and constructed in-house and described in detail elsewhere.^9^ This instrument holds a cylinder and piston assembly containing a RTIL sample, where the cylinder is metal (stainless steel), and the piston is made of Delrin and contains a center metal electrode. The cylinder assembly can be disassembled to insert the ITO-coated glass or modified ITO-coated glass discs, which function as the cylinder head. The unreacted ITO disc (reference) and the surface-modified ITO-coated disc (sample) are inserted and 200 μL of C_4_PyTFSI is added to the assembled cylinder. The seal between the cylinder and piston is made using a Buna-N O-ring. The resulting seal allows air to escape but seals the RTIL in place upon the application of force.^7,22^ The device is a second class lever that allows access to forces up to ∼4500 N.^7^ The piezoelectrically generated current transients are measured as a function of applied force using an electrometer (Keithley 6517A) controlled using a LabVIEW VI written in-house. The force applied is measured using a calibrated digital force gauge (Nidec model FG-3009).

The manner in which we measure the direct piezoelectric response is not compatible with controlling the surface charge of the RTIL support in the same manner as was done for the converse piezoelectric *ρ*_*f*_ measurements, a factor that complicates the ability to make the comparison of interest. For the converse piezoelectric effect measurements, we controlled the surface charge of the ITO support by passing electric current through the support surface to control the steady state carrier (*e* ^*–*^) density, an arrangement that is incompatible with our measurement system for the direct piezoelectric effect. To overcome this issue, we have controlled the support surface charge chemically for the direct piezoelectric effect measurements.

Achieving such control is not a simple matter owing to the chemistry of the ITO surface. The ITO coating on a glass support typically exhibits a complex grain structure where the density of reactive sites is not well characterized and can vary with the manner of surface preparation. To overcome this issue, we have chosen to modify the ITO surface by the deposition of a monolayer of modified graphene oxide. The characteristic lateral extent of the graphene oxide sheets are well in excess of the average distance between reactive sites on a bare ITO surface, and the thickness is ∼2.3 nm. We formed graphene oxide from sheets of graphene that were prepared in our laboratory. The graphene oxide sheets were reacted with POCl_3_ and H_2_O to produce the phosphated-graphene oxide (P-GO) material. This material has been found to be reproducible in terms of the achievable density of functionalities.^17^ The deposited P-GO monolayer serves to remap the ITO surfaces with a reproducible spatial distribution of phosphate functionalities that yield an interface with a net negative surface charge. Changing the charge on the P-GO surface from negative to positive is achieved by reacting the exposed -OPO_3_^2–^ groups with Zr^4+^. The density and distribution of charged sites thereby remains the same for both the positively- and negatively charged supports, and gaining this control over interfacial charge is accomplished with a monolayer of nanometer thickness.^17^

As with any measurement involving surfaces, water plays an essential if ill-characterized role. Even though C_4_Py TFSI contains less than 50 ppm water (by KF analysis), it takes very little adventitious water to interact with the comparatively small number of functionalized surface sites. For the -OPO_3_^2–^ surfaces, interaction of the phosphate functionalities with water will be fundamentally different than it is for the Zr^4+^ terminated surfaces, making direct comparison of the two supports potentially problematic. It is this chemical variability that is likely responsible for the sample-to-sample variations in the I vs F data (Figure 3). To overcome this issue, we have compared the direct piezoelectric response of C_4_Py TFSI in contact with both modified surfaces to the response of this RTIL in contact with unreacted ITO surfaces. We thus compare the direct piezoelectric responses of the -OPO_3_^2–^ and Zr^4+^ terminated surfaces to that of the ITO coated surface for each measurement set, to provide an internal standard.

**Figure 3:**
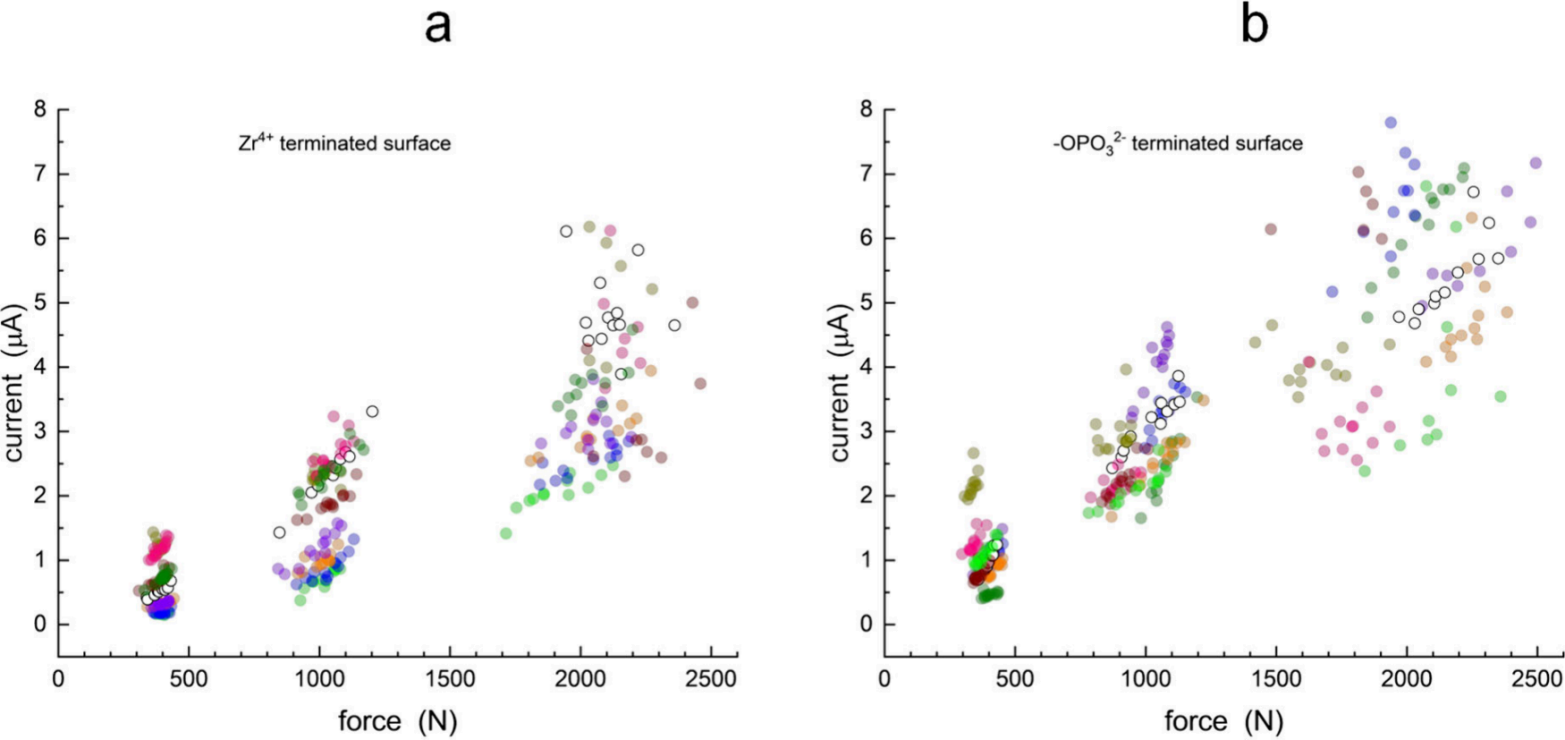
Relationship between the transient current and force applied for C_4_Py TFSI on (a) Zr^4+^-terminated support and (b) -OPO_3_^2–^-terminated support. Data for the unreacted ITO support (open circles) are provided in both panels. Eight sets of the P-GO modified ITO discs were evaluated. The colors indicate data for individual samples (data for each run is presented individually in Figures S1–S8).

We show the transient current vs applied force data for the positively charged surface (Figure 3a) and the negatively charged surface (Figure 3b), and the unreacted ITO surface (open circles in both figures). Eight samples sets of positively and negatively charged surfaces (prepared simultaneously for each set) were measured to gauge the reproducibility of the results. The data show that the positively charged supports produces a slope for the I vs F data that is lower than that for the unreacted ITO support, while the negatively charged support produces I vs F data with a slope that is essentially the same as for the unreacted support. There are several interesting features contained in these data. The data are clustered in distinct groups of applied force, which reflects the application of force at three distinct positions on the lever of the instrument, with variations within each cluster being determined by shot-to-shot variation in the applied force. While there is some vertical variation between the individual data sets, it is the slopes of the I vs F data that are important. The intercepts for the I vs F data are expected to vary with the background current for each individual sample, which is determined by the presence of adventitious conductive species, such as water.

To understand the difference between the direct piezoelectric responses C_4_Py TFSI in contact with the Zr^4+^- and -OPO_3_^2–^-terminated supports, we compare in Figure 4 the slopes of the I vs F data shown in Figure 3 for each sample examined. The slopes of the data for C_4_Py TFSI in contact with the modified surfaces are normalized to slopes for the same RTIL in contact with an unreacted ITO surface. While the ratios vary somewhat from sample-to-sample, in all cases the direct piezoelectric response of C_4_Py TFSI in contact with the -OPO_3_^2–^ surface is larger than for the Zr^4+^ surface. The average slope for C_4_Py TFSI response for the Zr^4+^-terminated support normalized for the unreacted ITO support is 0.74 ± 0.28 and the average for the -OPO_3_^2–^ support is 1.20 ± 0.40. While these values are not vastly different, they are measurably different, and for all eight samples, the Zr^4+^-terminated support yields a lower C_4_Py TFSI response than the -OPO_3_^2–^-terminated support. There is thus a surface-charge-based difference in the direct piezoelectric response of C_4_Py TFSI, which correlates with the behavior seen for the converse piezoelectric response of RTILs.

**Figure 4:**
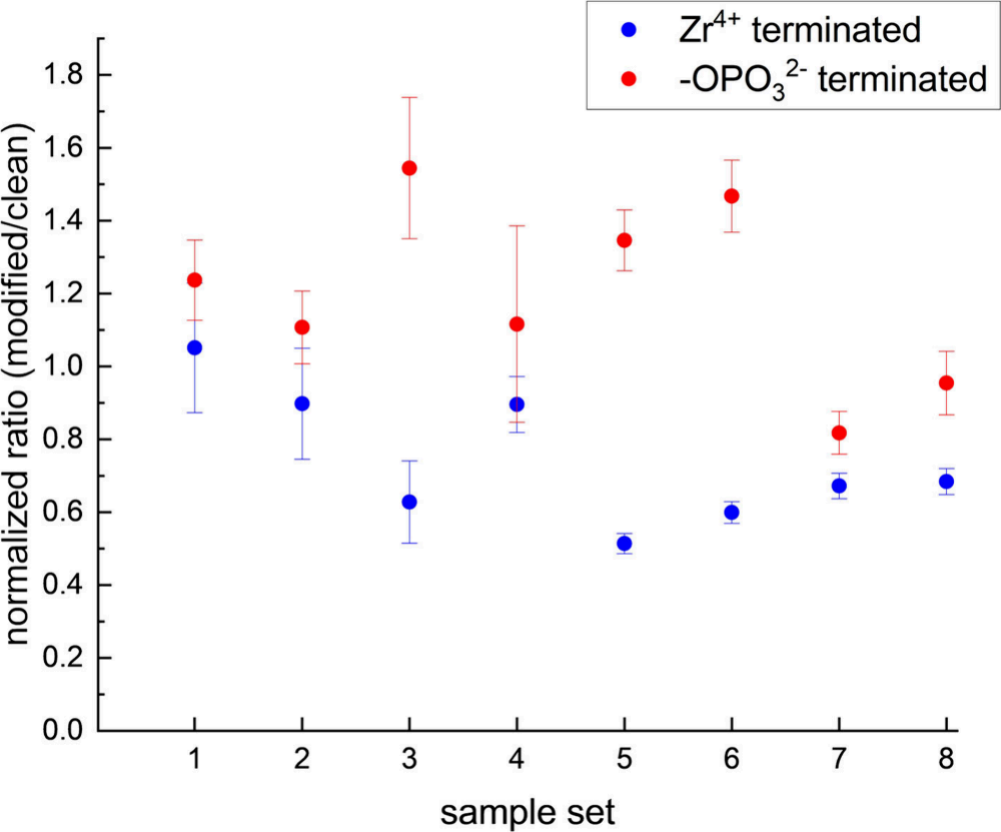
Normalized data for the direct piezoelectric response of C_4_Py TFSI in contact with Zr^4+^-terminated and -OPO_3_^2–^-terminated supports for eight sample sets. A sample set is a pair of ITO-coated supports modified simultaneously, with one sample reacted with Zr^4+^ and the other left terminated with phosphate functionality. Data are normalized to the response of C_4_Py TFSI in contact with an unreacted ITO support. Error bars are propagated from the uncertainties (±1σ) of the best fit slopes of the regressed data for each sample set (data shown in Figures S1–S8).

We consider these results in the context of what is currently known about interface-induced organization in RTILs. This organization has been observed in terms of structural order and charge density gradients, and the characteristic length scales of each appear to not correspond directly. Anaredy et al.^11,23^ and Lasar et al.^13^ have reported direct structural evidence for interface-induced order in RTILs, with the evolution time of the order being comparatively slow (minutes to hours). The persistence length of this order can be up to approximately one μm,^11,23^ and depending on the RTIL cation and anion structures, domain sizes on the order of 400 to 700 nm have been reported.^13^ While this latter domain size seems to correlate with earlier estimates of transient pressure-induced crystal dimensions,^9^ it remains to be established if there is an actual structural correlation between the domains seen in the liquid phase and transient crystalline solids responsible for the direct piezoelectric response.

The induced charge density gradient, *ρ*_*f*_, reported by the Blanchard group has a characteristic persistence length of ∼50 μm.^14,16,24–29^ The detection and characterization of *ρ*_*f*_ was made using rotational diffusion measurements of charged chromophores, where the gradient was observed through depth-dependent variations in the concentrations of RTIL cations and anions giving rise to changes in the relative fractions of free and associated chromophores. While this gradient is seen to modulate the dielectric response of the RTILs,^29^ it is not possible to infer whether there is a detectable structural gradient within the RTIL. It was also found that the establishment of *ρ*_*f*_ in the RTILs was rapid (on the order of seconds at most), in contrast to the establishment of structural order over ∼1 μm length scales.^11,13,23^ It is possible that *ρ*_*f*_ serves to poise the liquid phase in a manner favorable to the formation of crystalline species upon the application of pressure. Much work remains to be done to place the relationship between the direct and converse piezoelectric effects in RTILs on a more solid structural footing, but the surface charge-dependence of the direct piezoelectric response in C_4_Py TFSI reported here is consistent with *ρ*_*f*_ poising the liquid phase RTIL in different ways depending on the sign of the surface charge.

We have reported the surface charge dependence on the direct piezoelectric response of the room-temperature ionic liquid C_4_Py TFSI. This surface charge-dependence, while modest, correlates with the known dependence of *ρ*_*f*_ on the sign of surface charge and thus establishes a new connection between the converse and direct piezoelectric responses of RTILs. Further work will be needed to establish the relationship(s) between *ρ*_*f*_, interfacial organization and the direct piezoelectric response in RTILs, and this work serves as a foundational effort in the development of nanoscale liquid phase piezoelectric sensing and piezopneumatic actuation applications.

## Supplementary Material



## Notes

Individual data sets for the relationship between the transient current and force applied for C_4_Py TFSI on Zr^4+^-terminated support and -OPO_3_^2–^-terminated support shown in Figure 3 (DOCX)
