# Supplementary material for: Surface Charge Dependence of the Direct Piezoelectric Response of a Room Temperature Ionic Liquid: Implications for Nanoscale Control over Sensing and Actuation
Source: ACS Appl Nano Mater. 2026 Apr 1;9(14):6051–5. doi: 10.1021/acsanm.6c00579 (PMC13077625; doi:10.1021/acsanm.6c00579)
Supplement: Supplementary file 1 [file an6c00579_si_001.docx]

Supporting Information

Surface Charge Dependence of the Direct Piezoelectric Response of a Room Temperature Ionic Liquid: Implications for Nanoscale Control over Sensing and Actuation

Neelanjana Mukherjee, Sheryl S. Blanchard and G. J. Blanchard^^[[1]](#footnote-1)^*^

Michigan State University, Department of Chemistry

578 S. Shaw Lane, East Lansing, MI 48824 USA

**Figures S1 – S8**. Individual data sets for the data shown in Figure 3. Left: Filled circles are I vs. F data for a Zr^4+^-terminated surface. Open circles are I vs. F data for a bare ITO support. Right: Filled circles are I vs. F data for a -OPO_3_^2-^ -terminated surface. Open circles are I vs. F data for a bare ITO support.

**Figure S2**. Left: Filled circles are I vs. F data for a Zr^4+^-terminated surface. Open circles are I vs. F data for a bare ITO support. Right: Filled circles are I vs. F data for a -OPO_3_^2-^ -terminated surface. Open circles are I vs. F data for a bare ITO support.

**Figure S1**. Left: Filled circles are I vs. F data for a Zr^4+^-terminated surface. Open circles are I vs. F data for a bare ITO support. Right: Filled circles are I vs. F data for a -OPO_3_^2-^ -terminated surface. Open circles are I vs. F data for a bare ITO support.

**Figure S4**. Left: Filled circles are I vs. F data for a Zr^4+^-terminated surface. Open circles are I vs. F data for a bare ITO support. Right: Filled circles are I vs. F data for a -OPO_3_^2-^ -terminated surface. Open circles are I vs. F data for a bare ITO support.

**Figure S3**. Left: Filled circles are I vs. F data for a Zr^4+^-terminated surface. Open circles are I vs. F data for a bare ITO support. Right: Filled circles are I vs. F data for a -OPO_3_^2-^ -terminated surface. Open circles are I vs. F data for a bare ITO support.

**Figure S6**. Left: Filled circles are I vs. F data for a Zr^4+^-terminated surface. Open circles are I vs. F data for a bare ITO support. Right: Filled circles are I vs. F data for a -OPO_3_^2-^ -terminated surface. Open circles are I vs. F data for a bare ITO support.

**Figure S5**. Left: Filled circles are I vs. F data for a Zr^4+^-terminated surface. Open circles are I vs. F data for a bare ITO support. Right: Filled circles are I vs. F data for a -OPO_3_^2-^ -terminated surface. Open circles are I vs. F data for a bare ITO support.

**Figure S8**. Left: Filled circles are I vs. F data for a Zr^4+^-terminated surface. Open circles are I vs. F data for a bare ITO support. Right: Filled circles are I vs. F data for a -OPO_3_^2-^ -terminated surface. Open circles are I vs. F data for a bare ITO support.

**Figure S7**. Left: Filled circles are I vs. F data for a Zr^4+^-terminated surface. Open circles are I vs. F data for a bare ITO support. Right: Filled circles are I vs. F data for a -OPO_3_^2-^ -terminated surface. Open circles are I vs. F data for a bare ITO support.

1. * Author to whom correspondence should be addressed: email: blanchard@chemistry.msu.edu, Tel: +1 517 353 1105 [↑](#footnote-ref-1)
